# Prevalence and associated factors of hypertension among veterans of the Indian Gorkha regiments living in Pokhara Metropolitan City, Nepal

**DOI:** 10.1186/s12913-021-06907-1

**Published:** 2021-09-01

**Authors:** Abhishek Sapkota, Dinesh Neupane, Aamod Dhoj Shrestha, Tara Ballav Adhikari, Craig Steven McLachlan, Naveen Shrestha

**Affiliations:** 1 Nepal Development Society, Chitwan, Nepal; 2 Johns Hopkins Bloomberg School of Public Health, Baltimore, MD USA; 3 Department of Public Health, Section for Global Health, Aarhus University, Aarhus, Denmark; 4 Health Faculty, Centre for Healthy Futures, Torrens University Australia, Adelaide, Australia; 5 CiST College, Pokhara University, Kathmandu, Nepal

**Keywords:** Hypertension, veterans, retired army, NCDs, occupational health, Nepal

## Abstract

**Background:**

Hypertension is a major preventable risk factor for cardiovascular disease. Occupational factors such as having served or serving in armed forces may be associated with hypertension. This study aimed to assess the prevalence and factors associated with hypertension among veterans of the Indian Gorkha army living in western Nepal.

**Methods:**

A community-based cross-sectional study was conducted among the veterans living in the Pokhara metropolitan city. Data on blood pressure (BP), anthropometric measurements, and behavioral factors were collected by face-to-face interviews using the World Health Organization's non-communicable disease risk factor surveillance (STEPS) tool. Hypertension was defined as systolic blood pressure (BP) ≥ 140 mm Hg and/or diastolic BP of ≥ 90 mm Hg or currently on antihypertensive medication.

**Results:**

The age-adjusted prevalence of hypertension was 66.2 % among the study participants (317). Mean systolic and diastolic blood pressure was 144.5 mmHg (± 18.3) and 89.3mmHg (± 16.0), respectively. Among the hypertensive participants, 67 % were aware of their disease, 90 % of them were under treatment, and 14 % of the individuals who received treatment had their hypertension under control. The proportion of smokers was 12.9 % and alcohol drinker was 86.1 %. One-fourth (25.9 %) of the participants had a family history of hypertension. Veterans aged 55-64 years had higher odds (AOR: 5.3; 95 % CI: 1.8–15.9; *p* = 0.003) of being associated with hypertension as compared to 35–44 years. Being a current alcohol drinker (AOR: 2.5; 95 % CI: 1.4–4.5; *p* = 0.003), overweight (AOR: 1.9; 95 % CI: 1.0-3.5; *p* = 0.04), obese (AOR: 3.1; 95 % CI: 1.1–8.3; *p* = 0.03) and family history of hypertension (AOR: 2.9; 95 % CI: 1.5–5.8; *p* = 0.002) were independently associated with hypertension.

**Conclusions:**

Hypertension was prevalent in retired Nepal veterans. Hypertension was associated with a number of modifiable lifestyle and behavioral factors. Our findings suggest the need for screening, education and management of Nepal veterans for hypertension.

## Background

Globally, hypertension is a significant risk factor for cardiovascular disease (CVD) [1]. The age-standardized global prevalence of hypertension is 31.1 % [2, 3]. It is estimated that 18 % of adults in the low- and middle-income countries (LMICs) are hypertensive, of which two-thirds remain untreated [4]. In South Asia, after Afghanistan, Nepal has the highest burden of hypertension with an estimated prevalence to be circa 28 % [5, 6]. Occupational factors can influences long term stress responses that can result in hypertension. For example, having served or serving in the armed forces maybe associated with increased risk for developing hypertension [7, 8]. In the United States of America (USA), protective service occupations had a significantly increased prevalence of hypertension compared to management occupations when controlling for age. Furthermore, 51 % of veterans have been reported to be hypertensive [9]. Similarly, an analysis of 20 years’ data of American veterans showed that 42 % of them developed CVDs [10]. Nearly 54 % of retired army officers in China were found hypertensive, whereas only 20.9 % of service officers (non-retired) had hypertension [11].

There are limited studies assessing the prevalence of hypertension among army veterans in Nepal. A study conducted among war veterans in eastern Nepal showed a 35.6 % prevalence of raised blood pressure among veterans, and the odds of being hypertensive was higher compared to farmers and labors [7]. It is unknown whether Indian army veterans in Nepal have similar levels of hypertension as reported in other countries. Our aim is to estimate the prevalence of hypertension and associated factors among veterans of the Indian Gorkha army living in Pokhara Metropolitan City, of Western Nepal.

## Methods

### Study design and study population

A community-based cross-sectional study was conducted in Pokhara metropolitan City. The study included veterans of the Indian Gorkha army who are permanent residents of the Pokhara metropolitan city.

### Sample size calculation and sampling procedure

The required sample size of the study was 317, determined by assuming 20% prevalence of hypertension [12], allowable error (α) of 0.05, design effect of 1.2, and confidence interval of 95%. Assuming a response rate of 90%, the sample size was raised to 348 for this study. The sampling frame was obtained by contacting the chairperson responsible for each retired group of veterans, across 6 localities of Pokhara metropolitan city. Each veteran was assigned a number in chronological order. Then, 348 veterans were selected using lottery method. Regarding the number of young participants, the minimum age required to be eligible for the Indian army is 18 years, and they can retire after 18 years in service. Only a few of the military officers retire after 18 years. Those who retire at young age leave the country to work as security officers in foreign country. So, only few of the young participants were available at the time of the study.

### Data collection procedure

Face to face interview and physical measurements wereconducted at the respondents’ households. The study tool was adapted from the World Health Organization’s validated STEPS tools version 3.1 [13], which have been validated in Nepali by Nepal Health Research Council (NHRC) [14]. Anthropometric measurements were taken using standardized techniques and calibrated equipment.

### Blood pressure measurement

Blood pressure was measured using a digital measuring device (Dr. Morepen Model BP-09) with participants sitting after resting for at least fifteen minutes. Three BP measurements were taken at five-minute intervals. The average of the last two measurements was included in the analysis. Hypertension was defined as a systolic BP of ≥ 140 mm Hg and/or diastolic BP of ≥ 90 mm Hg or the use of antihypertensive medication [15].

### Socio-demographic variables

Socio-demographic variables included in the study were age group in years (35–44, 45–54, 55–64, > 64), and ethnicity, which was categorized into upper caste, Janajatis, and others according to the caste coding by the Government of Nepal [16].

### Behavioral variables

Current smoker was defined as smoking at least one cigarette in the last 30 days and current alcohol consumption was defined as at least one drink in the last 30 days. Low physical activity was defined as those who scored less than 3,000 metabolic equivalents of the task of moderate or vigorous activities per week, while those with more than 3,000 metabolic equivalents of the task were defined as adequate physical activity [17].

### Anthropometric measurements

Weight was measured using a digital scale, and height was measured using a portable stature scale. Body mass index (BMI) was calculated by dividing weight in kilogram by square of height in meters. BMI was classified as underweight (< 18.5 kg/m^2^), normal (≥ 18.5 kg/m^2^ to < 25 kg/m^2^), overweight (≥ 25 kg/m^2^ to < 30 kg/m^2^) and obesity (≥ 30 kg/m^2^). Waist and hip circumferences were measured using John’s non-stretchable measuring tape. As all participants were male, participants with waist circumference ≥ 90 centimeters were defined to have central obesity [18].

### Awareness, treatment, and control of hypertension

 Participants who reported that a physician ever told them they had hypertension were considered aware of their hypertensive conditions. Participants receiving any kind of anti-hypertensive drugs in the last two weeks were categorized as undergoing treatment [19]. Control of hypertension was defined as hypertensive participants having an average systolic BP < 140 mm Hg and an average diastolic BP < 90 mm Hg [19].

### Data analysis

The collected data were checked for consistency and completeness and were entered in EpiData version 3.1(The EpiData Association, Odense, Denmark) and analyzed using IBM SPSS Statistics for Windows, version 20.0(Armonk, NY: IBM Corp). Frequency distribution, mean and percentage were calculated as descriptive analysis. A multiple logistic regression analysis was performed with hypertension as the dependent variable and age, sex, ethnicity, smoking, alcohol consumption, physical activity, BMI, central obesity, and family history of hypertension as the independent variables. Those variables with a p-value ≤ 0.05 were considered statistically significant associations.

## Results

### Socio-demographic characteristics

A total of 317 veterans participated in the study, with a response rate of 91 %. All were male between 39 and 92 years of old. The mean age (± SD) was 62 (± 11.8) years. Table 1 shows the socio-demographic and behavioral characteristics of the study participants. The majority of the participants were above 64 years (40.1 %) and Janajati (87.4 %). The proportion of current smokers, current alcohol drinkers, low physical activity, family history of hypertension, overweight and central obesity were 12.9 %, 55.5 %, 18.6 %, 25.9 %, 51.4 %, and 6.6 %, respectively.

**Table. 1:** Socio-demographic and behavioral characteristics of the study participants

Characteristics (*n* = 317)	Frequency	Percentage
**Age**		
35–44 years	20	6.3
45–54 years	73	23
55–64 years	97	30.6
> 64 years	127	40.1
**Ethnicity**		
Upper caste	38	12
Janajati	277	87.4
Others	2	0.6
**Currently Smoking**		
Yes	41	12.9
No	276	87.1
**Current alcohol consumption**		
Yes	176	55.5
No	141	44.5
**Physical activity**		
Low	59	18.6
Adequate	258	81.4
**Family History of Hypertension**		
Yes	82	25.9
No	235	74.1
**BMI**		
Underweight	2	0.6
Normal	107	33.8
Overweight	163	51.4
Obese	45	14.2
**Central Obesity**		
Yes	21	6.6
No	296	93.4

### Prevalence of Hypertension

The mean (SD) systolic and diastolic BP readings were 144.5 (18.3) and 89.3(16.0) mmHg, respectively. The age-adjusted prevalence of hypertension was 66.2 %. Among all individuals identified as hypertensive, 67 % were aware of their condition. Nearly 90 % of those aware of being hypertensive were receiving treatment. However, less than one-fifth of the treated subjects had their hypertension under control (14 %).

### Factors associated with Hypertension

Table 2 presents the association of hypertension with different risk factors. Veterans aged 55–64 years had higher odds (AOR: 5.3; 95 % CI: 1.8–15.9; *p* = 0.003) of being associated with hypertension as compared to 35–44 years. Being a current alcohol drinker (AOR: 2.5, 95 % CI: 1.4–4.5; *p* = 0.003), overweight (AOR: 1.9, 95 % CI: 1.0-3.5; *p* = 0.04), obese (AOR: 3.1, 95 % CI: 1.1–8.3; *p* = 0.03) and family history of hypertension (AOR: 2.9, 95 % CI: 1.5–5.8; *p* = 0.002) were independently associated with hypertension. Factors such as ethnicity, current smoking, physical activity, and central obesity were not significantly associated with hypertension.

**Table. 2:** Association between hypertension and major risk factors

Risk factors (*N* = 317)	Hypertension		OR(95 %CI)	*p*-value	AOR (95 % CI)	*p*-value
	**Yes(%)**	**No (%)**				
**Age (in years)**						
35–44 years	10 (50)	10 (50)	Ref			
45–54 years	41 (56.1)	32 (43.8)	1.3 (0.5–3.5)	0.6	1.5 (0.5–4.6)	0.44
55–64 years	74 (76.3)	23 (23.7)	3.21 (1.2–8.7)	0.02	5.3 (1.8–15.9)	**0.003**
> 64 years	91 (71.7)	36 (28.5)	2.52 (0.9–6.6)	0.06	5.5 (1.8–16.6)	**0.002**
**Ethnicity**						
Upper caste	28(73.7)	10(26.3)	Ref			
Janajati	187(67.5)	90(32.5)	0.7(0.4–1.6)	0.44	0.6 (0.2–1.4)	0.24
Others	1(50)	1(50)	0.4 (0.2–6.3)	0.48	0.3 (0.0 -5.7)	0.41
**Currently alcohol user**						
No	86(61)	55(39)	Ref			
Yes	130(73.9)	46(26.1)	1.8 (1.1–2.9)	0.02	2.5 (1.4–4.5)	**0.003**
**Current smoker**						
No	188(68.1)	88(31.9)	Ref			
Yes	28(68.3)	13(31.7)	1.0 (0.5-2.0)	0.98	1.0 (0.4–2.4)	0.96
**Physical activity**						
Adequate	174(67.4)	84(32.6)	Ref			
Low	42(71.2)	17(28.8)	1.2 (0.6–2.2)	0.58	1.8 (0.9–3.8)	0.13
**Body Mass Index (Kg/m2)**						
Normal	63(58.9)	44(41.1)	Ref			
Underweight	1(50)	1(50)	0.7 (0.0-11.5)	0.80	2.8 (0.1–64.8)	0.52
Overweight	116(71.2)	47(28.8)	1.7 (1.0-2.9)	0.04	1.9 (1.0-3.5)	**0.04**
Obese	36(80)	9(20)	2.8 (1.2–6.4)	0.02	3.1 (1.1–8.3)	**0.03**
**Central obesity (cm)**						
No	199(67)	97(33)	Ref			
Yes	17(81)	4(19)	2.1 (0.7–6.3)	0.20	1.5 (0.4–5.4)	0.53
**Family history of hypertension**						
No	149(63.4)	86(36.6)	Ref			
Yes	67(81.7)	15(18.3)	2.6 (1.4–4.8)	0.003	2.9 (1.5–5.8)	**0.002**

## Discussion

This study found the age-adjusted prevalence of hypertension to be 66.2 % in army veterans of the India-Gorkha regiment residing in Pokhara Metropolitan city of Western, Nepal. In addition, this prevalence was higher than that of previous study among war veterans of Nepal that showed a prevalence of 35.6 % in 2007 [7]. This prevalence is higher than the WHO global report and other population-based studies in Nepal [3, 12, 20, 21]. We acknowledge that our age groups, ethnicity, and occupation of participants are different than the WHO report.

From a global perspective, the prevalence of hypertension in our study is higher than a meta-analysis on prevalence rates of major chronic diseases in retired and in-service Chinese military officers. This meta-analysis explored 75 articles with a sample size of 90,758, which showed an overall prevalence of 53.8 % among retired officers, while it was 20.9 % among in-service officers [11].

In our study, risk factors such as being a current smoker, inadequate physical activity, central obesity, and hypertension were prevalent in higher age groups, i.e., 55–64 years. Similar cardiovascular risk factors for hypertension have been previously reported in Nepal [22]. Higher age, alcohol consumption, overweight, obesity, and family history of hypertension were found to be associated with hypertension in our study. These risk factors associated with hypertension are similar for the general population in Nepal [22, 23].

The awareness rate and treatment rate of hypertension in our study were similar to a study conducted among retired troops in China which showed an awareness rate of 45.1 % and a treatment rate of 94.6 % [24]. However, the rate of hypertension control was high, with 62.3 % [24].On the other hand, the rate of control in our study was only 14 %.

There may be some explanations for the prevalence of hypertension to be high in our studies. Firstly, we have focused on individuals in armed service after retirement. As opposed to younger active recruits may be fit and have lower hypertension rates [25]. Indeed, with aging, one would expect an increase in systolic blood pressure with aortic stiffening, particularly with smoking or alcohol intake. There was also a significant increase in BMI in those that were hypertensive as compared to those that were not. Obesity and or metabolic syndrome are risk factors once again for hypertension [26]. While there was a trend towards reduced fitness and hypertension, this was not statistically significant, despite a large component of the cohort being overweight. Finally, while not assessed, active combat can be associated with post-traumatic stress disorder that is significantly associated with increased risky behaviors such as smoking and alcohol intake which can increase cardiovascular risk [27].

The strengths of our study are a random sampling of participants, validated tools, and the measurement of blood pressure using WHO guidelines. The study also had various limitations. Firstly, the study was confined to a limited number of wards of Pokhara metropolitan. However, a reasonable sample size was obtained. Secondly, as the study was cross-sectional, hence a causal relationship between hypertension and socio-demographic and behavioral variables cannot be inferred. Thirdly, there might have been some bias in self-reported behaviors such as tobacco use, alcohol use, and physical activity. Lastly, it is acknowledged that a number of risk factors can influence hypertension development, such as additional environmental factors, however in our study we have focused on commonly reported risk factors that are modifiable for cardiovascular risk.

## Conclusions

Our study concludes that the age-standardized prevalence of hypertension is high among veterans of the Indian Gorkha regiments living in Pokhara Metropolitan City. Furthermore, hypertension was found to be significantly associated with age, current alcohol use, family history of hypertension, and BMI. Behavioral interventions targeting modifiable risk factors such as obesity, smoking, and alcohol intake may be effective in reducing the burden of hypertension. 

## Data Availability

The data used to support the findings of this study are available from the corresponding author upon reasonable request.
